# Appropriately Tuning Stochastic-Psychometric Properties of the Balloon Analog Risk Task

**DOI:** 10.3389/fpsyg.2022.881179

**Published:** 2022-05-10

**Authors:** Simone Di Plinio, Mauro Pettorruso, Sjoerd J. H. Ebisch

**Affiliations:** ^1^Department of Neuroscience, Imaging, and Clinical Sciences, G D’Annunzio University of Chieti-Pescara, Chieti, Italy; ^2^Institute for Advanced Biomedical Technologies (ITAB), G D’Annunzio University of Chieti-Pescara, Chieti, Italy

**Keywords:** BART, psychometrics, task optimization, risk-taking, computational neuroscience, stochasticity

## Abstract

The Balloon Analog Risk Task (BART) allows to experimentally assess individuals’ risk-taking profiles in an ecologically sound setting. Many psychological and neuroscientific studies implemented the BART for its simplicity and intuitive nature. However, some issues in the design of the BART are systematically unconsidered in experimental paradigms, which may bias the estimation of individual risk-taking profiles. Since there are no methodological guidelines for implementing the BART, many variables (e.g., the maximum explosion probabilities, the rationale underlying stochastic events) vary inconstantly across experiments, possibly producing contrasting results. Moreover, the standard version of the BART is affected by the interaction of an individual-dependent, unavoidable source of stochasticity with a trial-dependent, more ambiguous source of stochasticity (i.e., the probability of the balloon to explode). This paper shows the most appropriate experimental choices for having the lowest error in the approximation of risk-taking profiles. Performance tests of a series of simulated data suggest that a more controlled, eventually non-stochastic version of the BART, better approximates original risk-taking profiles. Selecting optimal BART parameters is particularly important in neuroscience experiments to optimize the number of trials in a time window appropriate for acquiring neuroimaging data. We also provide helpful suggestions to researchers in many fields to allow the implementation of optimized risk-taking experiments using the BART.

## Introduction

The administration of psychometric tests is a crucial phase in scientific investigations aimed at understanding human behavior, including cognitive and behavioral individual differences, predispositions toward clinical and subclinical symptoms, and the neurocognitive framework supporting psychometric variability (Lenhard et al., 2019; Zickar, 2020; Tonini et al., 2021). To achieve such aims, it is crucial that the statistical-mathematical structure and the psychological-philosophical background of psychometric instruments are sound, unbiased, and consistent (Nichols et al., 2017; Steiner and Frey, 2022). Ideally, reproducible simulations should test task performance variations for their psychometric and stochastic properties, including their general efficiency in measuring latent behavior (Schonberg et al., 2011; Bajracharya and Duboz, 2013; Donkin et al., 2017; Fitzpatrick, 2019).

The Balloon Analog Risk Task (BART) is a valuable laboratory-based psychometric instrument to assess risk-taking behavior in healthy and clinical populations. From its initial development (Lejuez et al., 2002), the BART has been used in many experiments, producing a large body of literature. The widespread utilization of the BART is motivated by its capability in recreating an ecological experience to uncover (neuro)cognitive underpinnings of risk-taking in healthy subjects (Lejuez et al., 2002, 2003, 2005; Weafer et al., 2011; De Groot, 2020; Guenther et al., 2021). However, measurements of risk-taking behavior are also interesting for clinical research since risk-taking indexed by BART scores has been associated with dysfunctional psychophysiological phenotypes, including anxiety (Maner et al., 2007; Buelow and Barnhart, 2017), clinical disorders (Hunt et al., 2005; Swogger et al., 2010; Dominguez, 2011; Cheng et al., 2012; Robbins et al., 2012; Reddy et al., 2014; Brown et al., 2015; Fischer et al., 2015; Tikàsz et al., 2019; Boka et al., 2020; Luk et al., 2021), abuse of heavy drugs (Hopko et al., 2006; Vassilva and Conrod, 2019), smoking attitudes (Lejuez et al., 2003, 2005; Dean et al., 2011; Hanson et al., 2014), alcohol consumption and related symptoms (Skeel et al., 2008; Fernie et al., 2010; Ashenhurst et al., 2011; Weafer et al., 2011; DeMartini et al., 2014; King et al., 2014), gambling (Holt et al., 2003; Mishra et al., 2017), risky sexual behavior (Lejuez et al., 2004; Bornovalova et al., 2008; Lawyer, 2013; for reviews on risk-taking and related dysfunctions see: Leigh, 1999; Turner et al., 2004; Isles et al., 2019). Moreover, BART use has been suggested as a potential marker for dissecting disease-related endophenotypes (Long et al., 2020).

The experimental design of the BART is intuitive and straightforward (Lejuez et al., 2002). In every trial, a balloon is presented, and the participant is asked to either take an award proportional to the current balloon size or attempt inflating the balloon to increase the value of the award. Every time the participant chooses to inflate the balloon, it can be inflated or explode. The explosion of the balloon implies that the award for the current trial is zero (or a negative value). For its simplicity and ecological validity, the BART has been used in neuroimaging studies (Rao et al., 2008; Cazzell et al., 2012; Sela et al., 2012; Congdon et al., 2013; Helfinstein et al., 2014; Xu et al., 2016; Guo et al., 2018; De Groot and Strien, 2019) and showed reliability for both behavioral and neural responses (White et al., 2008; Li et al., 2020).

Although the underlying paradigm is reasonably practical, the BART in its standard design entails unsolved criticalities that may hinder the usefulness of the collected data. Previous works discussed putative issues of the BART, including the censoring of information, the confusion of risk with the expected value, a poor distinguishing between uncertainty and risk, and the ambiguity in the definition of adaptive versus maladaptive behavior (Schonberg et al., 2011; De Groot and Thurik, 2018; De Groot, 2020; Canning et al., 2022). While these concerns are relevant, a significant source of inaccuracies in the BART is the unsafe interaction between two stochastic processes. First, individual uncertainty and noisy behavior are non-avoidable sources of stochasticity. In other words, there is a certain degree of uncertainty in subjective choices, which is subjective and driven by the context and the outcomes generated in previous trials, which blends with noisy behavior (Schonberg et al., 2011; Isles et al., 2019; Yakobi and Danckert, 2021). Consequently, this first stochasticity is a mix of noise and useful subject-specific information. Second, implementing random consequences of individual choices (inflating vs. explosion) means decreasing experimental control over the paradigm and introducing an additional source of stochasticity. We refer to these two sources of stochasticity as “individual-dependent stochasticity” and “design-dependent stochasticity,” respectively. Since the impact of the design-dependent stochasticity has not been appropriately investigated yet, the present study explicitly addresses the design-dependent stochasticity in the BART.

In this study, we show that the power of the BART to reconstruct individual risk-taking profiles significantly raises if its methodological implementation is controlled. More specifically, controllable sources of stochasticity must be handled to achieve accurate experiments. To propose an unbiased, reproducible, and controlled version of the BART, we implement a series of simulations with varying parameters such as explosion probability, virtual risk-taking profiles, number of inflations, number of trials, number of subjects, noise. Importantly, varying these parameters likely allows to model the possible experimental variations of the BART and to test their relative efficiency. Findings from these simulations will therefore assist psychologists and other researchers in psychometric measurements of risk-taking. For example, an optimal paradigm will aid in situations in which researchers must improve their experimental paradigm due to time and instrumental limitations, like, for example, in the case of clinical neuroimaging experiments. The evidence reported by our investigation, together with the implementation of naturalistic and unbiased measures (Schmitz et al., 2016; Coon and Lee, 2021; Yakobi and Danckert, 2021; Steiner and Frey, 2022), will allow scientists to adopt the BART in the best possible way following their experimental demand.

## Materials and Methods

The great majority of studies implementing the BART linearly or exponentially modulate the probability of balloon explosion across consecutive inflations. Generally, the explosion probability is modulated to increase between 0 (0% explosion, the balloon will surely inflate) and 1 (100% explosion, the balloon will explode).

### Modeling Explosions in Multiple Variants of the BART

Conceptually, we implemented a series of simulations to control how explosion probabilities were modeled. We incorporated and expanded the variations used in past studies utilizing the BART to investigate the impact of the following parameters in the reconstruction of actual risk-taking profiles: the explosion probability maximum *threshold*; the *function* used to calculate monotonically increasing explosion probabilities across inflations; the number of inflation events & the number of trials; the number of virtual players (participants); the noise in the virtual players’ behavior; the implementation of non-stochastic (i.e., deterministic) explosions.

### Simulating Virtual Risk-Taking Profiles

**Figure 1:**
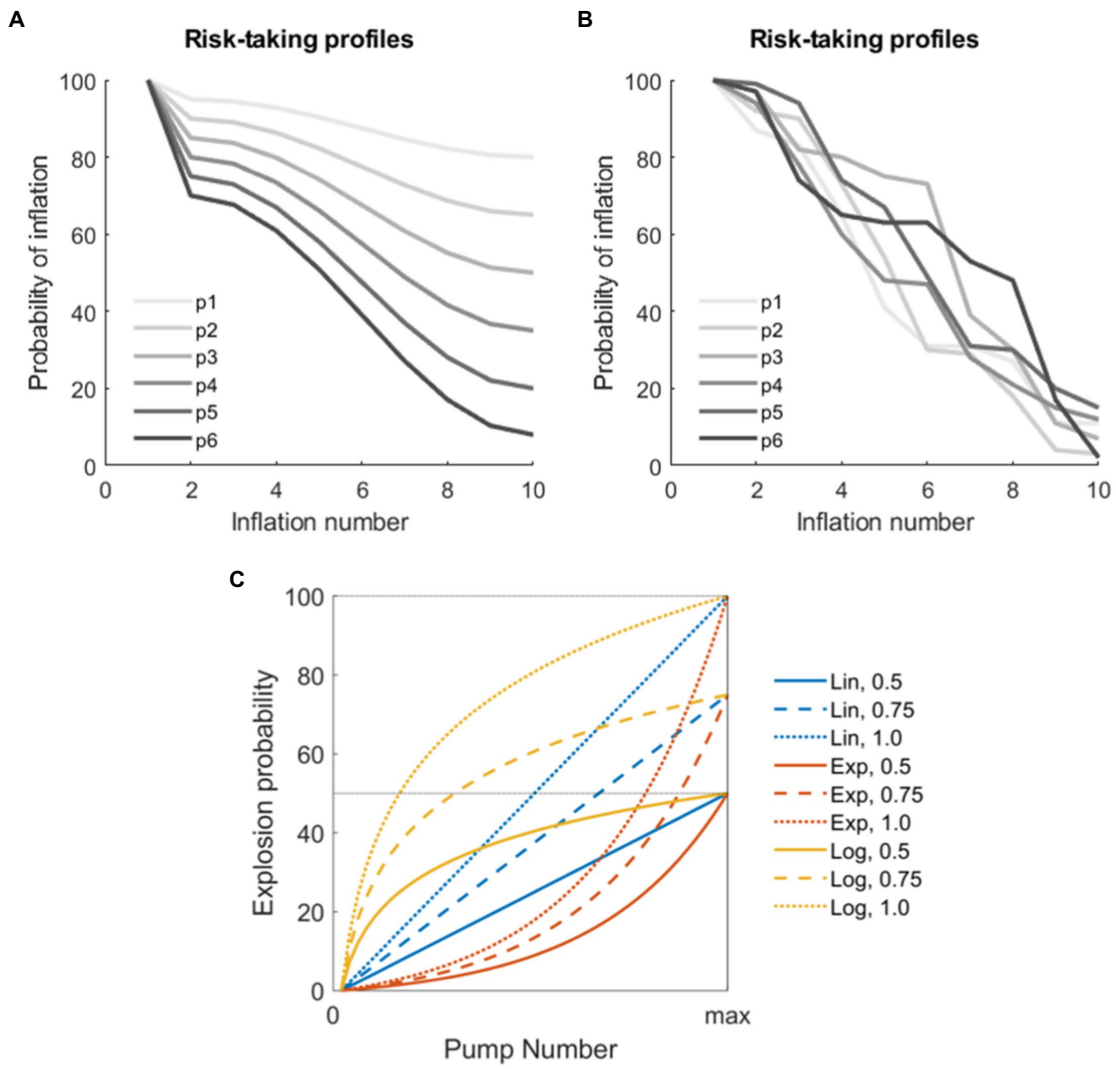
Parameters for the simulation. **(A)** Examples of virtual players with non-linear decrease in the risk-taking profile modeled using the cosine function. **(B)** Examples of virtual players with random decrease in the risk-taking profile. **(C)** Combination of parameters used to model explosions in the different versions of the stochastic Balloon Analog Risk Task (BART) analyzed. The three functions used to model explosion probabilities in consecutive inflations are coded by colors (linear, exponential, and logarithmic). The three thresholds indicating maximum explosion probabilities are coded by the line type (50, 75, and 100%).

We tested the performance of different BART versions using a series of simulations implemented in MatLab (The Mathworks, version 2019b). In these simulations, several risk-taking profiles were initially generated. Assuming N virtual subjects and T trials per subject, the simulation of risk-taking profiles ultimately produced, for each virtual subject *n*, and for each trial *t*, a series of K monotonically decreasing numbers comprised between 100 and 0 (where 100 = the virtual subject will surely try to inflate the balloon at this inflation event, and 0 = the virtual subject will surely choose the award). The parameters K, N, and T were varied according to different parameters (see below). We used two sets of risk-taking profiles for the simulations: the first set comprehended monotonically decreasing cosine functions starting from 100 and ending in a random number between 0 and 80; the second set was generated using random numbers varying between 0 and 100 reordered to represent monotonically decreasing functions. These simulated profiles were chosen since they approximate realistic risk-taking profiles from real subjects reported in the BART literature (Schonberg et al., 2011; Yakobi and Danckert, 2021), thus simulating both subjects with a regular behavior and subjects with an irregular (noisier) behavior. Example profiles are shown in Figures 1A,B.

### Simulating Decision Matrices

After generating individual risk-taking profiles, the BART was run for each subject, using T trials and a maximum of K inflation events. Before the actual simulation of the BART, an individual decision matrix was generated. That matrix was a T-by-K (trial-by-possible inflations) binary matrix indicating if the virtual subject chooses to inflate/take the prize for each possible event of the task. The decision matrix was generated as follows. In each trial, a decision of the *n*th virtual subject is simulated by extracting a random number between 1 and 100: if the random number is above the critical threshold for that *n*th subject, in the *t*th trial, and in the *k*th inflation event, then the corresponding decision for the current trial is set to “take the award”; alternatively, if the random number is below the critical threshold for that *n*th subject in the *k*th inflation event, then the corresponding decision for the current trial is set to “try inflating the balloon.” Importantly, modeling *exactly the same* degree of intra-subject variability (i.e., subject-related stochasticity) is mandatory to investigate the accuracies of different parametrizations of the BART. The decision matrix was simulated for each virtual subject (i.e., from each risk-taking profile) at each simulation cycle before running the BART itself. To note, this stage entailed the first, unavoidable source of stochasticity in the BART.

### The BART

The individual decision matrix was subsequently applied to the task. In each trial, the virtual decision is used to prompt the BART algorithm alternatively toward the “take the award” or “try inflating the balloon” choice. The “take award” option leads to obtaining a certain amount of money and going to the subsequent trial. Instead, the “try inflating the balloon” option led to the extraction of a random number between 1 and 100: if the random number was below the critical explosion threshold in the *k*th inflation event, in the *t*th trial, then the balloon exploded; alternatively, if the random number is equal or above the threshold, then the trial continues to the next inflation (*k*th + 1). This stage was modeled for three explosion probability functions: linear increase function, exponential increase function, and logarithmic increase function. Moreover, three maximum explosion thresholds were set: 50, 75, and 100%. These functions are represented in Figure 1C. To note, this stage represented the second source of stochasticity in the BART.

### Simulation Parameters

Investigating an increasing number of maximum inflation events (K) and trials (T) is crucial since it influences the psychometric properties of the BART (Lejuez et al., 2002; Fecteau et al., 2007; Rao et al., 2008). Furthermore, many neuroimaging experiments must comply with time and instrumental limitations, utilizing fewer trials in event-related designs. We selected a range of combinations of K and T to allow a comprehensive examination of the effect of double sources of stochasticity in the BART. Thus, K was tuned to have the values [6 10 16 24 48]. At the same time, the number of trials was varied systematically to investigate both scenarios with time limitations (e.g., neuroimaging studies or experiments with time limitations in general) and less coerced procedures (e.g., behavioral studies). Expressly, with a low number of inflations (*K* = 6, *K* = 10, *K* = 16), the total number of trials T was set to 48. Instead, with a high number of inflations (*K* = 24, *K* = 48), T was set to 150. We did not expect the number of subjects N to impact errors particularly. However, given the importance of combining trial number and sample size in neuroscience (Nichols et al., 2017; Chen et al., 2022), we investigated variable sample sizes in the current study, ranging from 20 to 100 virtual subjects [20, 50, 100]. Finally, we investigated two levels of noise in the virtual subjects: in the low-noise condition, each individual decision of the decision matrix was distorted by adding a random number in the range [−0.1 0.1]; instead, in the high-noise condition, each individual decision of the decision matrix was distorted by adding a random number in the range [−0.4 0.4].

### Non-stochastic BART

In the standard, stochastic BART (s-BART), each time the virtual participant attempted to inflate the balloon, it alternatively exploded or not depending on a random number. Instead, in the deterministic BART (d-BART), balloons were programmed to explode from a particular inflation event without the need for further unavoidable randomizations. In the d-BART, when the virtual subject attempted inflating the balloon (i.e., when the *k*th-by-*t*th element of the decision matrix was “try inflating”), it deterministically exploded or not based on the maximum number of inflations allowed for that balloon: if the max number of inflations was exceeded, then the balloon exploded; if not, then the trial continued to the next inflation event. We also implemented this d-BART in the simulations since it may be presumed that avoiding the second source of stochasticity may improve the estimation of risk-taking profiles.

### Statistics

Reconstructed profiles were averaged and plotted separately for each parametrization. The performance was assessed using linear mixed-effects regression models. The dependent variable was the estimation error (the difference between the real risk-taking profile and the reconstructed profile). Fixed effects were added for the factor threshold (three levels: 50, 75, and 100%) and the factor function (three levels: linear, exponential, and logarithmic). Random intercepts were added for each virtual *subject*, and random intercepts and slopes were added concerning the grouping factor *inflation* (e.g., for the analysis of a simulation with 10 inflation events, the grouping factor *inflation* had 10 levels). Thus, the model was in the Wilkinson form “error ~ threshold * function + (1|subject) + (threshold + function|inflation).” Secondly, we compared the best combination of threshold & function from the s-BART with the d-BART. The linear mixed-effects models were applied independently for each combination of trials, inflations, and noise levels. Linear contrasts within the model were used to directly compare appropriate levels of the fixed factors.

## Results

We ran each simulation scenario (i.e., each unique combination of parameters) 100 times separately for low and high noise levels. After each cycle, original risk-taking profiles were reconstructed for each virtual subject using each combination of parameters for modeling explosions (linear increase with 100% maximum threshold, linear increase with 75% maximum threshold, linear increase with 50% maximum threshold, exponential increase with 100% maximum threshold, exponential increase with 75% maximum threshold, exponential increase with 50% maximum threshold, logarithmic increase with 100% maximum threshold, logarithmic increase with 75% maximum threshold, logarithmic increase with 50% maximum threshold, deterministic BART).

**Figure 2:**
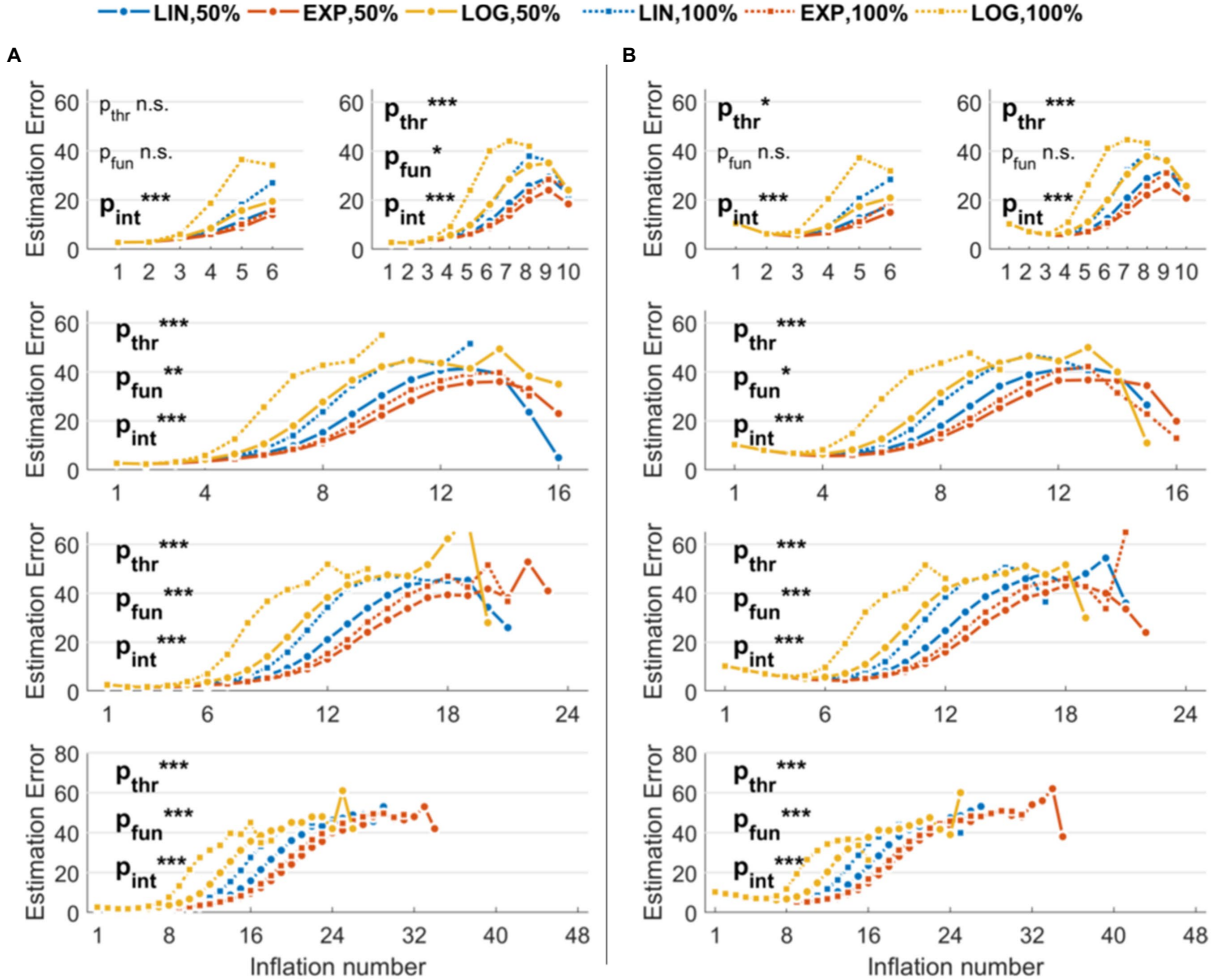
Estimation errors of the stochastic version of the Balloon Analog Risk Task (BART) implemented in the simulations. The three functions used to model explosion probabilities in consecutive inflations are coded by colors (linear, exponential, and logarithmic). The three thresholds indicating maximum explosion probabilities are coded by the marker and line type (only 50 and 100% thresholds are shown to improve readability). **(A)** Results with low noise levels in the virtual participants’ decisions. **(B)** Results with high noise levels in the virtual participants’ decisions. Legend: ^***^p < 0.001; ^**^p < 0.01; ^*^p < 0.05.

Figure 2A shows the performance of different parameter combinations with low levels of noise. The fixed factors and the interaction were significant in almost every situation, indicating an effect of both threshold and function used to model balloon explosion on the error in estimating original risk-taking profiles. More specifically, with six maximum inflations (*K* = 6), we found a trend for threshold (*p*_THR_ = 0.07), a non-significant effect for function (*p*_FUN_ = 0.49), and a significant interaction threshold-by-function (*p*_INT_ < 0.001). With higher *K* values, these effects were always significant (*K* = 10: *p*_THR_ < 0.001, *p*_FUN_ = 0.04, *p*_INT_ < 0.001; *K* = 16: *p*_THR_ < 0.001, *p*_FUN_ = 0.004, *p*_INT_ < 0.001; *K* = 24: *p*_THR_ < 0.001, *p*_FUN_ < 0.001, *p*_INT_ < 0.001; *K* = 48: *p*_THR_ < 0.001, *p*_FUN_ < 0.001, *p*_INT_ < 0.001). Linear contrasts of interest for the effects of interest are reported in Table 1 (center columns). The performance of different parameter combinations with high levels of noise are shown in Figure 2B. Also in this case, the fixed factors and the interaction effects were significant. More specifically, with six maximum inflations (*K* = 6), we found a significant effect of threshold (*p*_THR_ = 0.03), a non-significant effect for function (*p*_FUN_ = 0.47), and a significant interaction threshold-by-function (*p*_INT_ < 0.001). With 10 maximum inflations (*K* = 10), we found a trend for threshold (*p*_THR_ < 0.001), a trend for function (*p*_FUN_ = 0.05), and a significant interaction threshold-by-function (*p*_INT_ < 0.001). With higher *K*s, effects were always significant (*K* = 16: *p*_THR_ < 0.001, *p*_FUN_ = 0.03, *p*_INT_ < 0.001; *K* = 24: *p*_THR_ < 0.001, *p*_FUN_ < 0.001, *p*_INT_ < 0.001; *K* = 48: *p*_THR_ < 0.001, *p*_FUN_ < 0.001, *p*_INT_ < 0.001). Linear contrasts of interest for the effects of interest are reported in Table 1 (right columns).

**Table 1:** Direct contrasts showing the accuracy increase when using lower thresholds and exponential functions.

Inflations (trials)	Factor	Linear contrast of interest	Low noise		High noise	
			*β* (SE)	*p*-Value	*β* (SE)	*p*-Value
6 (48)	*Threshold*	75% vs. 50%	1.0 (0.6)	0.08	1.3 (0.7)	0.07
		100% vs. 50%	2.8 (1.4)	0.04^*^	3.2 (1.5)	0.04^*^
	*Function*	Lin vs. Exp	1.1 (1.0)	0.28	1.2 (1.0)	0.26
		Log vs. Exp	2.7 (1.4)	0.03^*^	2.9 (1.5)	0.01^*^
10 (48)	*Threshold*	75% vs. 50%	2.2 (0.7)	<0.001^*^	2.3 (0.7)	<0.001^*^
		100% vs. 50%	4.2 (1.2)	<0.001^*^	4.4 (1.2)	<0.001^*^
	*Function*	Lin vs. Exp	2.4 (1.2)	0.04^*^	2.5 (1.2)	0.04^*^
		Log vs. Exp	6.3 (1.5)	<0.001^*^	6.2 (1.5)	<0.001^*^
16 (48)	*Threshold*	75% vs. 50%	2.4 (0.5)	<0.001^*^	2.3 (0.5)	<0.001^*^
		100% vs. 50%	4.3 (0.8)	<0.001^*^	3.8 (0.8)	<0.001^*^
	*Function*	Lin vs. Exp	3.1 (1.1)	0.004^*^	2.8 (1.1)	0.01^*^
		Log vs. Exp	7.7 (1.5)	<0.001^*^	6.8 (1.6)	<0.001^*^
24 (150)	*Threshold*	75% vs. 50%	2.9 (0.5)	<0.001^*^	2.5 (0.5)	<0.001^*^
		100% vs. 50%	5.7 (0.9)	<0.001^*^	4.8 (0.8)	<0.001^*^
	*Function*	Lin vs. Exp	4.3 (1.2)	<0.001^*^	4.5 (1.2)	<0.001^*^
		Log vs. Exp	9.4 (1.6)	<0.001^*^	9.2 (1.6)	<0.001^*^
48 (150)	*Threshold*	75% vs. 50%	2.3 (0.3)	<0.001^*^	2.1 (0.3)	<0.001^*^
		100% vs. 50%	4.5 (0.5)	<0.001^*^	3.7 (0.5)	<0.001^*^
	*Function*	Lin vs. Exp	4.6 (1.0)	<0.001^*^	3.9 (0.9)	<0.001^*^
		Log vs. Exp	8.3 (1.5)	<0.001^*^	5.9 (1.5)	<0.001^*^

To note, since the dependent variable in the mixed-effects models was the error in estimating original risk-taking profiles, which are expressed in a range [1–100], the estimates (*β*s) in the table represent the percentage of improvement. For example, if the contrast “100% vs. 50%” is significant with a *β* = 4.3, it means that using a maximum threshold of 50% increases the accuracy in reconstructing original risk-taking profiles by 4.3%. As shown in the table, using lower thresholds and exponential functions generally increases accuracy independently on the maximum number of inflation events and the noise. Asterisks (*) mark significant results (*p* < 0.05).

**Figure 3:**
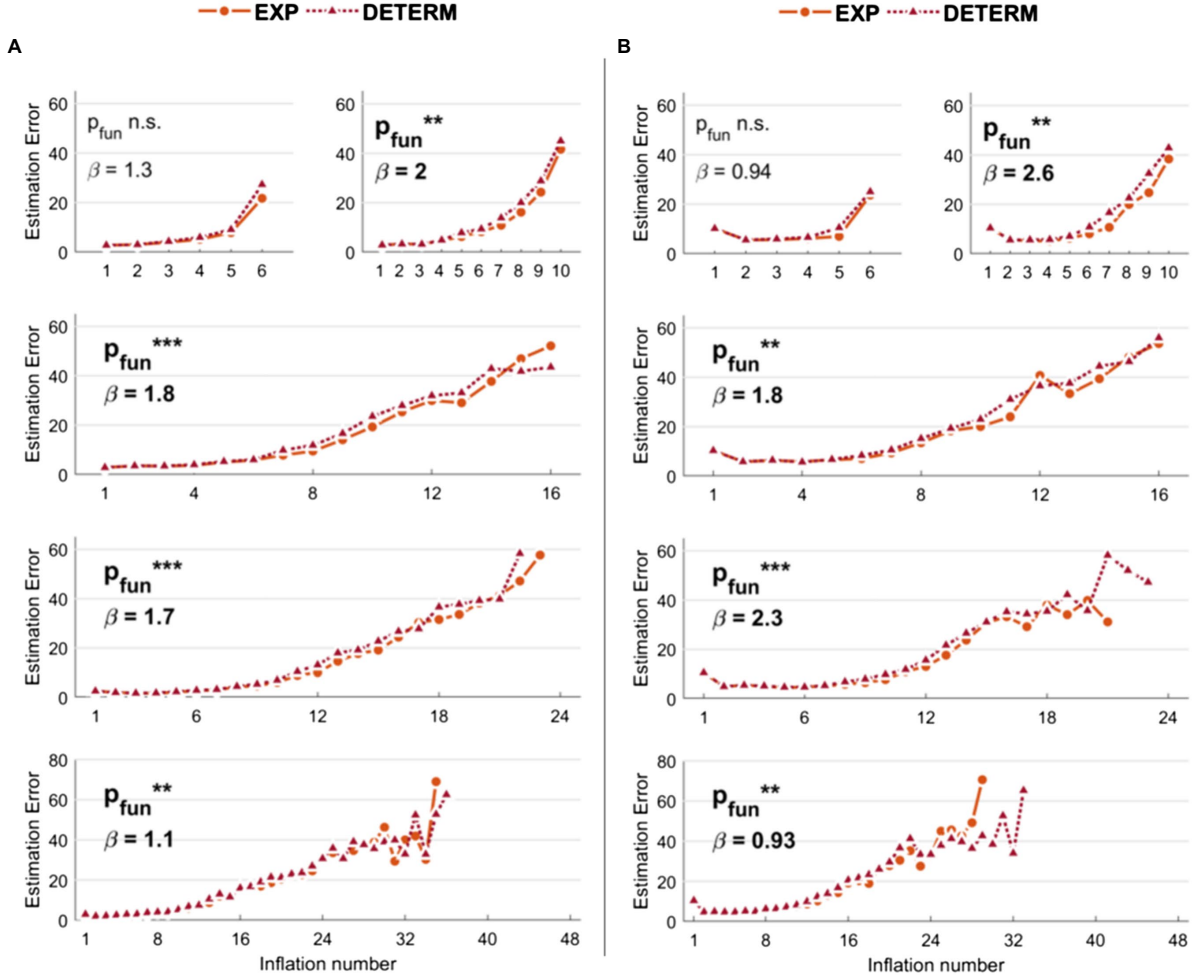
Estimation errors of the stochastic versus deterministic Balloon Analog Risk Task (BART) implemented in the simulations. With respect to stochastic BART, only results relative to explosions modeled with exponential function and 50% threshold are shown for comparison. **(A)** Results with low noise levels in the virtual participants’ decisions. **(B)** Results with high noise levels in the virtual participants’ decisions. Legend: ^***^p < 0.001; ^**^p < 0.01; ^*^p < 0.05.

Results related to the comparison of the s-BART versus d-BART are shown in Figure 3A (low noise levels) and 3B (high noise levels). For simplicity, we reported the comparison between the best s-BART model (i.e., 50% threshold and exponential explosion probability) and the d-BART model. Although the differences are smaller than in the previous models, it can be observed that the s-BART slightly outperforms the d-BART. With *K* = 6, the effect was not significant neither with low (*p* = 0.12) or high noise (*p* = 0.06). Instead, with higher number of inflations, the stochastic exponential and low threshold (50%) BART always outperformed the deterministic BART with both low noise levels (*K* = 10: *p* = 0.006; *K* = 16: *p* < 0.001; *K* = 24: *p* < 0.001; *K* = 48: *p* = 0.002) and high noise levels (*K* = 10: *p* = 0.002; *K* = 16: *p* = 0.001; *K* = 24: *p* < 0.001; *K* = 48: *p* = 0.009).

These results demonstrate that both the threshold and the function used to model stochastic explosions in the BART affect the performance in approximating original risk-taking profiles, independently on the noise level, maximum number of inflation events, and number of trials. Moreover, a crucial effect of the noise levels on the risk-taking profile estimation is appreciable: higher noise levels impact the approximation of information relative to the risk-taking profiles in earlier inflation events (first half), while the difference between high-noise and low-noise simulations is reduced in later inflations (second half). Finally, we show that the stochastic (standard) modeling of explosion probabilities is better than the deterministic modeling. The results reported here entail simulations with players’ virtual risk-taking profiles modeled using monotonically decreasing functions generated using random numbers, with *N* = 50. However, such effects are equivalent when varying the number of players (*N* = 20 and *N* = 100) and using monotonically decreasing functions with an exponential decrease to model virtual players’ risk-taking profiles.

## Discussion

Our study investigates a theoretical-methodological issue in one of the most frequently employed psychometric instruments to assess the risk propensity of individuals: the Balloon Analog Risk Task (Lejuez et al., 2002). We investigated the relationship between forms of stochasticity and expected psychometric measurements in experiments involving the BART. Two types of stochasticity coexist in this task: the first one reflects an informative variability related to individual uncertainty and noise (De Groot and Thurik, 2018; De Groot, 2020; Yakobi and Danckert, 2021); the second one arises when the experimental design is not appropriately controlled and theoretically does not convey any helpful information on risk-taking profiles.

We demonstrate that controlling the stochastic trial parametrization in the BART leads to a better approximation of the original risk-taking profiles. We show that using exponential functions to model explosion probabilities reduces estimation errors up to 9–10% for the original virtual risk-taking profiles. Moreover, using lower thresholds (maximum probability of explosions) reduces estimation errors up to 4–5%. Interaction effects in our models demonstrate that lower maximum thresholds of explosion probabilities (50 and 75%) combined with exponential functions for modeling stochastic explosions are the best combination to allow an efficient estimation of true risk-taking profiles. These results are consistent across many prior profiles (virtual participants) and across noise levels in the dataset. Finally, we demonstrate that the optimized version of the stochastic BART (s-BART) has a slightly better performance than the non-stochastic, deterministic BART (d-BART) although only the d-BART allows the complete control of the trial structure.

Given our findings, we recommend designing BART experiments to maximize the valuable information gathered from participants. A good choice would be to use a deterministic version of the BART with a maximum number of inflations between 10 and 16 or, alternatively, a stochastic version of the BART in which explosion probabilities are modeled using an exponential function ranging from 0 (first inflation) to 50% or 75%. This would allow gathering a sufficient number of trials even with a short administration (e.g., 10 min). Employing a version of the BART in which more inflation events are allowed (more than 20 inflations) may be helpful for a fine-grained resolution of risk-taking profiles only if there are enough trials (and time).

Suboptimal designs may have biased the approximation of individual risk-taking behavior in previous studies (see also Kılıç et al., 2020; Yakobi and Danckert, 2021), as some studies already suggested (Bishara et al., 2009; Purcell et al., 2017; De Groot and Thurik, 2018; Canning et al., 2022). For example, Maner et al. (2007) reported effects of anxiety on risk-taking, while a later study did not find such association (Buelow and Barnhart, 2017). King et al. (2014) reviewed the associations between impulsivity and alcohol consumption and observed discrepancies among studies that involved the BART. Some studies showed the BART to predict impulsivity related to alcohol abuse (Fernie et al., 2010; Rose et al., 2014) or to be unrelated with impulsivity (Skeel et al., 2008; Rieser et al., 2019). Moreover, DeMartini et al. (2014) found BART scores to correlate positively with the quantity of alcohol consumption but negatively with the frequency of alcohol consumption, while Ashenhurst et al. (2011) found that higher BART scores negatively correlate with alcohol-related symptoms but are unrelated to alcohol use. The literature about risky decision-making in clinical conditions is hard to interpret considering associations with BART scores: whereas some studies reported abnormal behavioral BART scores in people with schizophrenia (Dominguez, 2011; Reddy et al., 2014; Brown et al., 2015; Boka et al., 2020), other studies did not reproduce such results (Fischer et al., 2015; Tikàsz et al., 2019; Luk et al., 2021). At the same time, studies demonstrated both positive (Reddy et al., 2014) and negative (Dominguez, 2011) associations with symptom severity, while another study did not find associations (Cheng et al., 2012).

A limitation of the present study is that we did not simulate the influence of expected return on the risk-taking dynamics, so that we were not able to include losses versus zero-gain in case of balloon explosions (Schonberg et al., 2011; De Groot and Thurik, 2018; De Groot, 2020; Canning et al., 2022). In other words, our results can be interpreted as stemming from individuals with no differences regarding the impact of the expected win on risk-taking behavior. More in general, a simulation scenario like ours does not efficiently incorporate subjective priors and their eventual modification throughout the execution of the task. However, our results indicate that exponential functions and lower thresholds for balloon explosion provide a better estimation of behavioral profiles. This effect is presumably independent of the effect of expected values on behavior. Thus, our findings likely hold as demonstrated by our investigation of multiple simulated risk-taking profiles. It is also helpful to mention that the non-stochastic d-BART implies a more stringent selection of the number of trials compared to the s-BART. When selecting the number of trials (T) for the d-BART, T must always be a product of the maximum number of inflations. For example, with eight maximum inflation events, the total number of trials is 64, with each class of explosion repeated six times (8*6 = 64).

Since researchers can manipulate numerous facets of each experimental paradigm, even simple experimental procedures may have infinite applicative variants. However, choosing the proper experimental parameters is not always straightforward and intuitive. Researchers in all disciplines, including cognitive scientists, psychologists, and neuroscientists, should confidently select the most appropriate and unbiased experimental settings to generate reliable data and consequently make rational and safe claims (Schonberg et al., 2011; Bajracharya and Duboz, 2013; Donkin et al., 2017; Fitzpatrick, 2019; Chen et al., 2022). Our findings show how to optimize the reconstruction of original risk-taking profiles by allowing the extraction of the optimal amount of information through the administration of the BART. According to our findings, we suggest to model explosion probabilities using exponential monotonic increases and using a threshold (maximum explosion probability) between 50 and 75%. By limiting stochasticity unrelated with subject-specific information, our findings are particularly meaningful for the implementation of the BART in neuroimaging studies and for the investigation of clinical and subclinical phenotypes. Future clinical research will benefit from the improvement of this psychometric instrument for detecting aberrant decision-making processes, thus allowing to accurately monitor the efficacy of treatment targeting the pathophysiology of risky behaviors (Pettorruso et al., 2021). The accurate extraction of neurocognitive profiles will hopefully help guide clinicians in the selection of more personalized interventions. Researchers interested in applying this psychometric instrument to study risky behavior in both healthy and clinical population will greatly benefit from this design.

## Data Availability Statement

The original contributions presented in the study are included in the article/supplementary material; further inquiries can be directed to the corresponding author.

## Author Contributions

SD contributed to conception and design of the study, performed the statistical analysis, and wrote the first draft of the manuscript. SE supervised the study. All authors contributed to manuscript revision, read, and approved the submitted version.

## Conflict of Interest

The authors declare that the research was conducted in the absence of any commercial or financial relationships that could be construed as a potential conflict of interest.

## Publisher’s Note

All claims expressed in this article are solely those of the authors and do not necessarily represent those of their affiliated organizations, or those of the publisher, the editors and the reviewers. Any product that may be evaluated in this article, or claim that may be made by its manufacturer, is not guaranteed or endorsed by the publisher.
